# Management of ectopic Cushing syndrome in medullary thyroid carcinoma: from case reports to systematic literature review

**DOI:** 10.3389/fendo.2026.1933394

**Published:** 2026-09-09

**Authors:** Laura-Semonia Stanescu, Dumitru Ioachim, Andrei Muresan, Romeo Smarandache, Sofia-Maria Lider-Burciulescu, Monica Livia Gheorghiu, Corin Badiu

**Affiliations:** 1 “Carol Davila” University of Medicine and Pharmacy, Bucharest, Romania; 2 Pathology Department, “C. I. Parhon” National Institute of Endocrinology, Bucharest, Romania; 3 Research Department, “C. I. Parhon” National Institute of Endocrinology, Bucharest, Romania; 4 Surgery Department, “C. I. Parhon” National Institute of Endocrinology, Bucharest, Romania; 5 “Ana Aslan” National Institute of Geriatrics and Gerontology, Bucharest, Romania

**Keywords:** ectopic Cushing syndrome, hypercortisolism, medullary thyroid carcinoma, RET mutation, Selpercatinib, tyrosine kinase inhibitors

## Abstract

**Background:**

Ectopic Cushing’s syndrome (ECS) secondary to medullary thyroid carcinoma (MTC) is a rare endocrine manifestation associated with aggressive disease and considerable morbidity from severe hypercortisolism.

**Methods:**

We conducted a systematic review of PubMed and Web of Science from inception to January 25, 2026, in accordance with PRISMA guidelines, to identify all published cases of ECS due to MTC. Data were extracted on clinical presentation, RET mutational status, treatments, outcomes, and causes of death. In addition, we described four previously unreported patients from “C. I. Parhon” National Institute in Bucharest, Romania, analyzed independently from the systematic review dataset.

**Results:**

The systematic review identified 99 published cases of ECS caused by MTC. The mean age at diagnosis was 46.3 years (range 12–84), with male predominance (69.7%). Among cases with available genetic data, most were sporadic 32/43 cases (74.4%), frequently harboring somatic RET M918T mutation. MTC stage at diagnosis was available in 90 cases, of which 48 patients (53.3%) presented with distant metastases. ECS and MTC were diagnosed concomitantly in 45/89 patients (50.6%), while MTC preceded ECS in 43/89 patients (48.3%), with a median delay of 42 months. Hypercortisolism manifestations predominated and often masked locoregional thyroid disease. Thyroid surgery was the initial therapeutic approach in 62/77 cases (80.5%). Hypercortisolism was difficult to control and often refractory to multiple treatment strategies. Bilateral adrenalectomy was required in 33 patients, including 12 in whom it served as rescue treatment following failure of medical therapy. Selective RET inhibitors, particularly selpercatinib, have recently emerged as a promising treatment option, with all five reported cases demonstrating rapid biochemical improvement and radiologic responses. Among cases with available outcome data, mortality was primarily driven by complications of uncontrolled hypercortisolism and progressive metastatic MTC. Complementing the systematic review, regarding the four cases from our center, we have observed substantial disease burden, with thyroid surgery performed in every patient. Two cases received vandetanib producing favorable biochemical (calcitonin) and radiologic responses. Hypercortisolism proved challenging to manage, with three patients failing to achieve remission despite multimodal therapy, including surgery, targeted therapy, adrenalectomy, or medical treatment.

**Conclusion:**

ECS secondary to MTC is an exceptionally rare and highly morbid manifestation that occurs predominantly in metastatic sporadic MTC. Management of both tumor burden and hypercortisolism is complex and often requires multimodal therapy. Emerging data suggests that selective RET inhibitors may lead to simultaneous control of both tumor progression and cortisol excess in RET-mutated disease, although current data remain limited to case reports. The four institutional cases offer additional real-world clinical context and are presented separately from the systematic review to maintain methodological rigor.

## Introduction

Medullary thyroid carcinoma (MTC) is a neuroendocrine tumor originating from the parafollicular C cells of the thyroid and accounts for approximately 5% of all thyroid cancers (1). When detected early, MTC typically follows an indolent course, with 10-year overall survival rates approaching 95% for localized disease, declining to 75% with regional involvement, and approximately 40% in the presence of distant metastases (2, 3). Although disease stage at diagnosis is the primary prognostic factor, additional variables including age, hereditary versus sporadic subtype, and serum biomarkers such as calcitonin and carcinoembryonic antigen (CEA), contribute significantly to risk stratification (4, 5).

MTC-associated ectopic Cushing’s syndrome (ECS) is exceptionally rare, occurring in <1% of cases. In these patients, hypercortisolism results from ectopic secretion of corticotropin-releasing hormone (CRH) or adrenocorticotropic hormone (ACTH) by neoplastic C-cells (6, 7). ECS itself accounts for only 5–10% of ACTH-dependent CS and most commonly arises from small-cell lung carcinoma or other neuroendocrine tumors of the lung, thymus, or pancreas (8).

Although pro-opiomelanocortin (POMC) expression and ACTH immunoreactivity have been documented in 20%-100% of MTCs in historical cases, only a small subset produces clinically significant hormone excess (9, 10). Experimental studies indicate that only 2%–3% of immunoreactive ACTH in primary MTC cultures is biologically active, with tumor extracts dominated by high–molecular-weight “big ACTH” forms. These findings suggest impaired proteolytic processing of POMC (11). Thus, transcriptional activation alone is insufficient for a functional corticotroph phenotype.

The coexistence of MTC and ECS carries substantial clinical importance, as hypercortisolism drives significant morbidity and mortality and often signals aggressive tumor behavior. Effective management requires simultaneous control of the underlying malignancy and cortisol excess, using strategies such as surgical debulking, steroidogenesis inhibitors, bilateral adrenalectomy (BLA) in refractory cases, and, more recently, tyrosine kinase inhibitors (TKIs), which demonstrate benefit in addressing both tumor progression and ectopic hormone secretion (12). More recently, TKIs, including multi-targeted agents such as vandetanib and cabozantinib, and selective RET inhibitors such as selpercatinib, have demonstrated rapid biochemical remission of ectopic hypercortisolism alongside tumor control in RET-mutated MTC cases (12–14).

Given the exceptional rarity of ECS due to MTC, the available information was limited to isolated case reports and small case series. The present study provides a systematic review of ECS secondary to MTC, summarizing incidence, clinical presentation, therapeutic approaches, causes of death, and follow-up patterns. We also describe four cases from our institution to further illustrate the heterogeneity and diagnostic challenges of this entity.

### Additional institutional cases presentation

#### Case 1

A 70-year-old woman was referred to our Endocrinology department for evaluation of metastatic MTC, diagnosed by biopsy of a left axillary lymph node. She reported marked fatigue, proximal muscle weakness and significant dyspnea.

The diagnosis of metastatic MTC was strongly supported by markedly elevated calcitonin and CEA levels of 177000 pg/mL (normal value [n.v.] <5) and 715 ng/mL (n.v. <3), respectively. Endocrine work-up revealed ACTH-dependent Cushing’s syndrome: morning serum cortisol (S-cortisol) was 1600 nmol/L (n.v. 132.4-537.9), ACTH was 156 pg/mL (n.v. 3-66), and cortisol following 1 mg overnight dexamethasone suppression was 1186 nmol/L.

Whole-body computed tomography (CT) demonstrated multiple right-lobe thyroid masses, extensive cervical lymphadenopathy, pulmonary and hepatic metastases, and marked tracheal compression from the invasive thyroid tumor, explaining her respiratory symptoms. Magnetic resonance imaging (MRI) of the pituitary gland was unremarkable. There was no family history of MTC, and genetic testing for germline RET mutations was negative.

Airway compromise represented an immediate threat, and cytoreductive total thyroidectomy with bilateral central and lateral neck dissection was therefore prioritized for palliative airway relief. Histopathological examination confirmed MTC and immunohistochemical staining for ACTH was positive.

Following thyroidectomy, given the presence of metastatic disease, treatment with vandetanib was started (300 mg/day). One month after initiation, calcitonin decreased from a postoperative value of 82600 pg/mL to 70600 pg/mL (n.v. <5), and S-cortisol declined from 1034 nmol/L to 880 nmol/L (n.v. 132.4-537.9). Radiologic assessment performed four months after surgery demonstrated no new lesions and no progression of pulmonary and hepatic metastases.

After 12 months of treatment with vandetanib, imaging continued to demonstrate stable disease, and calcitonin levels further decreased to 43900 pg/mL (n.v. <5). However, hypercortisolism persisted, with morning S-cortisol measuring 882 nmol/L (n.v. 132.4-537.9), and the patient’s condition progressively deteriorated. Although cortisol lowering agents were recommended, they were not administered and bilateral adrenalectomy was not pursued given her frailty. Ultimately, the patient died of liver failure, a couple of months after last evaluation.

#### Case 2

A 53-year-old man was referred to our department because of a rapidly enlarging cervical mass. Recent medical history has been remarkable for hypertension and diabetes. Physical examination revealed a palpable mass in the right thyroid lobe, accompanied by dysphagia and dysphonia. He also had typical Cushingoid features, including central obesity with supraclavicular and dorsocervical fat accumulation, a round face, and proximal muscle atrophy and weakness.

Initial evaluation revealed elevated serum tumor markers, with calcitonin and CEA levels of 1815 pg/mL (n.v. <5) and 97 ng/mL (n.v. <3), respectively. Morning S-cortisol was 877 nmol/L (n.v. 132.4-537.9), 24-hour urine free cortisol >1590 µg/24h (n.v. 4.3–176) and ACTH was 115 pg/mL (n.v. 3-66), consistent with ACTH-dependent hypercortisolism. Furthermore, cortisol failed to suppress on the 8 mg dexamethasone suppression test (post-dexamethasone cortisol 827 nmol/L), supporting ectopic ACTH production.

CT imaging demonstrated a large right-lobe thyroid mass invading the trachea, esophagus, and adjacent vertebral bodies, and bilateral adrenal hyperplasia. Pituitary MRI revealed no lesion.

Although the patient had clinically overt ECS, he was clinically stable and the multidisciplinary team considered immediate surgery appropriate. Subsequently, the patient underwent complete thyroidectomy with bilateral central compartment and lateral compartment dissection. Histopathology confirmed MTC and immunostaining was positive for calcitonin and ACTH. Tumor genetic testing identified a somatic RET mutation, p.M1918T in exon 16.

After surgery, tumor markers decreased: calcitonin to 1290 pg/ml (n.v. <5) and CEA to 38 (n.v. <3), with normalization of cortisol levels 358 nmol/L (n.v. 132.4-537.9). Because the resection was incomplete due to extensive local invasion, further surgical intervention was not considered feasible, and systemic therapy with vandetanib was planned. However, initiation was deferred due to prolonged QTc intervals and ultimately commenced 8 months later, during which calcitonin and cortisol levels remained stable.

Six months after vandetanib initiation, serum calcitonin and CEA levels had markedly decreased to 37.8 pg/ml (n.v. <5) and 9.39 ng/ml (n.v. <3), respectively, while S-cortisol remained within the normal range. CT imaging demonstrated a 40% partial response according to RECIST 1.1 criteria. At the last follow-up, after 17 months of treatment with vandetanib, cortisol levels remained normal, and both biochemical and morphological assessments indicated stable MTC. The biochemical evolution over time is shown in.

#### Case 3

A man born in 1980 presented in 2000 after pituitary radiation therapy and bilateral adrenalectomy for presumed Cushing disease, with persistently elevated ACTH levels despite prior treatment. The medical history of this patient was scarce and no further information could be retrieved.

At presentation, he had progressively worsening diarrhea and generalized weakness. Laboratory evaluation revealed elevated ACTH of 88 pg/mL (n.v. 3-66), suppressed cortisol levels consistent with glucocorticoid replacement after adrenalectomy, and elevated calcitonin >900 pg/mL (n.v. <5), suggesting MTC as a likely source of ectopic ACTH production. CT imaging revealed a left-lobe thyroid nodule and a large mediastinal mass. Pituitary MRI was unremarkable.

During December 2001, the patient underwent mediastinal mass resection and total thyroidectomy. Histopathology confirmed metastatic MTC with positive calcitonin immunostaining. There was no family history of MTC or MEN 2/MEN3-related disorders. Also, RET genetic testing and systemic therapy with TKIs were not available in our country at that time.

After surgery, ACTH levels normalized, but diarrhea persisted. Octreotide provided only transient symptom relief for two months. Adjuvant external radiotherapy of the mediastinum was administered for the residual mass, without clinical or biochemical benefit.

At the last follow-up in September 2003, CT imaging demonstrated progression of the mediastinal mass and new pulmonary, and hepatic metastases. At that time, calcitonin was markedly elevated to >1,200 pg/mL (n.v. <5) and ACTH had risen to 257 pg/mL (n.v. 3-66). The patient was subsequently lost to follow-up.

#### Case 4

A 53-year-old woman presented with rapidly progressive clinical deterioration, including significant weight loss, proximal muscle weakness, dysphagia, and dyspnea. Laboratory evaluation revealed severe hypokalemia (2.1 mmol/L; n.v. 3.3-4.8). Endocrine work-up demonstrated ACTH-dependent Cushing’s syndrome, with elevated morning S-cortisol (1374 nmol/L; n.v. 184.8-623.4), ACTH (154 pg/mL; n.v. 3-66), and lack of suppression following the 8 mg overnight dexamethasone test (post-dexamethasone cortisol 5,633 nmol/L). Serum calcitonin was also markedly elevated (>2,000 pg/mL; n.v. <5), consistent with MTC.

CT imaging revealed an invasive right-lobe thyroid mass with cervical lymph-node, hepatic, pulmonary and bone metastases, bilateral adrenal hyperplasia, and a normal pituitary gland. Initial medical therapy with ketoconazole gradually escalated over two months to 800 mg/day, but serum cortisol concentrations did not decrease, and the patient’s condition deteriorated with marked hypokalemia. In this context, urgent bilateral adrenalectomy was considered as rescue treatment. At that time, RET genetic testing, and systemic therapy with TKIs were not available in our country.

One month after adrenalectomy, cytoreductive total thyroidectomy was performed and histopathology confirmed MTC. Three months after surgery, serum calcitonin was >2,000 pg/mL (n.v. <5) and ACTH had further increased to 393.2 pg/mL. The patient was subsequently lost to follow-up.

## Materials and methods

### Systematic review of literature

This systematic review was conducted following the Preferred Reporting Items for Systematic Reviews and Meta-Analyses (PRISMA) 2020 statement (15).

### Search strategy

A comprehensive literature search was performed in PubMed and Web of Science from database inception to January 25, 2026. The search strategy combined controlled vocabulary (MeSH terms) and free-text keywords related to MTC (“medullary thyroid carcinoma,” “medullary thyroid cancer”, “medullary carcinoma”, “medullary cancer”, “thyroid cancer”, “thyroid carcinoma”, “thyroid C-cell carcinoma”, “MTC”) and ECS (“Cushing syndrome”, “ectopic ACTH”, “ectopic Cushing syndrome”, “ectopic CRH”, “paraneoplastic Cushing syndrome”). Terms were combined using the Boolean operator AND. No language restrictions were applied at the search stage to maximize sensitivity and minimize selection bias. Reference lists of included articles and relevant reviews were hand-searched for additional eligible cases.

### Inclusion and exclusion criteria

All articles reporting cases of ECS due to MTC were included if they provided individual patient-level data with MTC confirmed on pathology and ACTH-dependent hypercortisolism attributable to MTC, defined by biochemical, imaging, or immunohistochemical evidence.

Articles were required to include sufficient clinical details to extract data on demographics, presentation, treatment, and outcomes. Studies were excluded if they reported unconfirmed MTC or ECS, described ECS due to non-MTC tumors, provided only aggregated data without extractable patient-level information, or lacked sufficient clinical details for data extraction. Non-English studies were excluded only when full-text translation was not feasible and abstracts lacked adequate clinical information.

### Study selection

**Figure 1:**
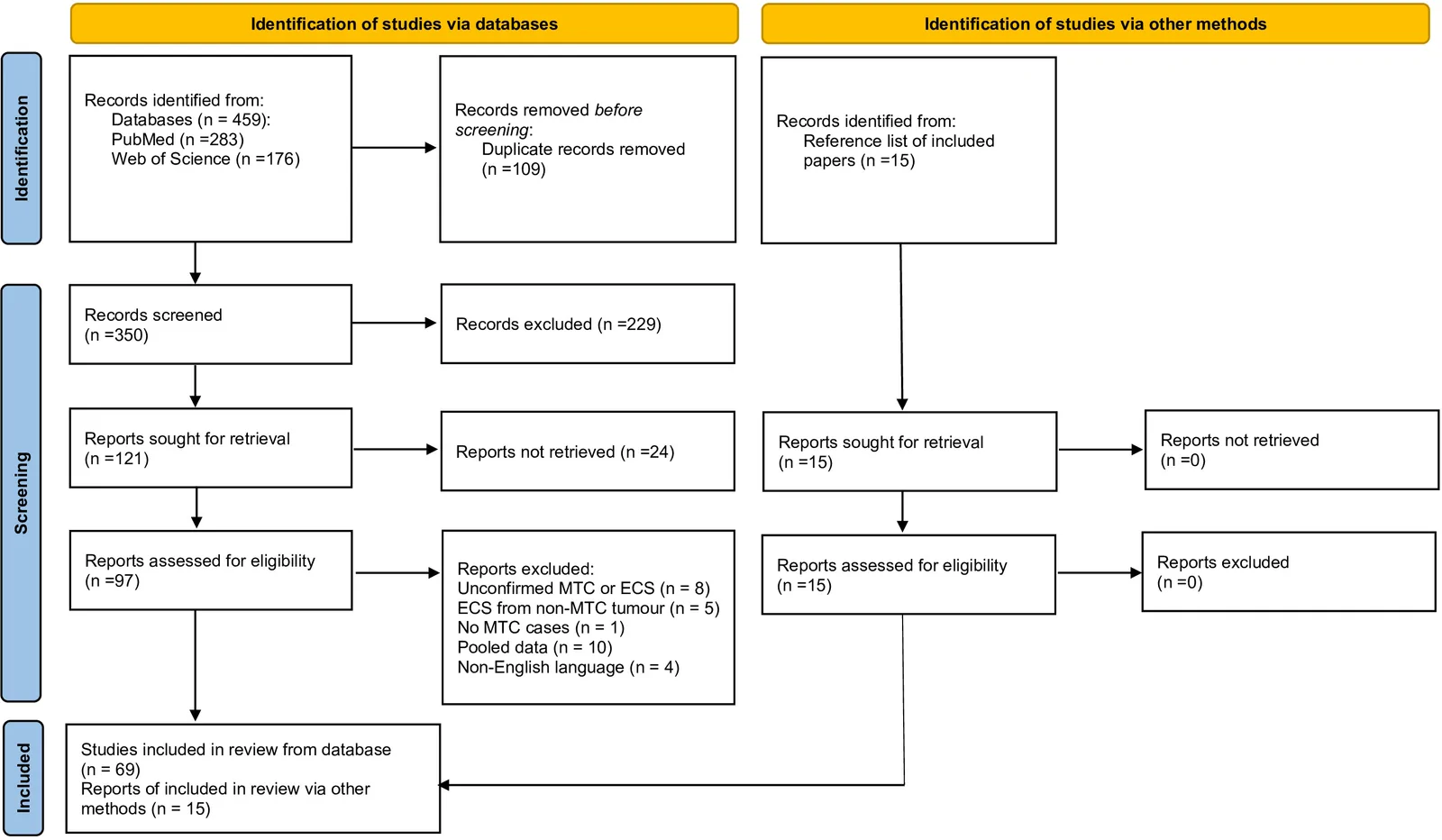
PRISMA 2020 flow diagram of the study selection process for the systematic review of ectopic Cushing syndrome secondary to medullary thyroid carcinoma.

All identified records were imported into a reference management system and duplicates removed. Titles and abstracts were screened, followed by full-text reviews of potential eligible articles. L.S.S. and C.B. independently performed study selection, with disagreements resolved by consensus or consultation with a third reviewer. The study selection process is summarized in the PRISMA 2020 flow diagram (**Figure 1**).

### Data extraction and quality assessment

Data was extracted using a chosen list of parameters, such as patient demographics, clinical features, treatments for both MTC and ECS, monitoring timelines, and outcomes. M.L.G. and S.M.L-B. independently extracted the data with discrepancies resolved through discussion. Given the rarity of the condition and significant heterogeneity in reporting, a narrative synthesis was performed. A formal quantitative quality-assessment tool was not applied, as the included literature consisted predominantly of case reports and small case series.

### Additional institutional cases

Four patients with MTC-associated ECS were compiled from the database used for the first author’s PhD thesis at ‘C. I. Parhon’ National Institute of Endocrinology, Bucharest, Romania. The thesis was approved by the Institutional Ethics Committee and Scientific Council (approval nr. 12/27.06.2022) and conducted in accordance with the Declaration of Helsinki (2013 revision). Written informed consent for inclusion in the database and publication of anonymized clinical data was obtained from all patients. These cases were not identified through the systematic literature search and were not included in the quantitative synthesis.

## Results

### Systematic review of literature

The search identified 459 articles, with an additional 15 identified through references checking. After removal of 109 duplicates, 365 articles underwent title/abstract screening; 257 were excluded as ineligible, leaving 108 full-text articles assessed for eligibility. Ultimately, 84 articles met inclusion criteria as references (16–94), also included in **Supplementary Table 2**, yielding 99 published cases of ECS secondary to MTC (**Figure 1**).

The mean age at diagnosis was 46.3 years (range 12–84 years), with a male predominance: 69/99 males (69.7%), and 30/99 females (30.3%). MTC subtype was specified in 43/99 cases (43.4%). Of these, 11/43 (25.6%) were hereditary (four MEN2, five MEN3 and two unclassified) and 32/43 (74.4%) sporadic cases (**Table 1**).

**Table 1:** Baseline characteristics of patients with medullary thyroid carcinoma and ectopic Cushing syndrome (Systematic Review, n=99).

Characteristics	No. (%)
Sex distribution	
Male sex	69/99 (69.7%)
Female sex	30/99 (30.3%)
Mean age at diagnosis, years (range)	46.3 (12-84)
MTC subtype	43/99 (43.4%)
Hereditary MTC	11/43 (25.6%)
MEN 2	4/11 (36.4%)
MEN 3	5/11 (45.4%)
Unclassified	2/11 (18.2%)
Sporadic MTC	32/43 (74.4%)
RET somatic mutation testing	9/32 (28.1%)
M918T mutation	6/9 (66.7%)
RET exon 16 mutation	1/9 (11.1%)
RET missense mutation	1/9 (11.1%)
No RET mutation	1/9 (11.1%)
Disease stage at diagnosis	90/99 (90.9%)
Distant metastases	48/90 (53.3%)
Locally advanced disease	19/90 (21.1%)
Regional lymph node metastases	21/90 (23.3%)
Probable metastatic disease	2/90 (2.2%)
Stage unavailable	9/90 (9.1%)
Timing: MTC vs ECS diagnosis	89/99 (89.9%)
Concomitant MTC and ECS	45/89 (50.6%)
MTC preceding ECS	43/89 (48.3%)
ECS preceding MTC	1/89 (1.1%)

ECS, Ectopic Cushing’s syndrome; MEN, multiple endocrine neoplasia; MTC, medullary thyroid cancer; RET, REarranged during Transfection proto-oncogene.

In the remaining cases, classification was not possible due to insufficient information, or cases were labeled “probably sporadic” or “probably hereditary” based on family/personal history (RET testing was unavailable in many older reports).

Among sporadic cases with somatic RET testing (n=9), 6 patients harbored the M918T mutation, one had a missense RET mutation in a liver metastasis, one had no RET mutations (germline and somatic), and one had a confirmed exon 16 mutation (**Table 1**).

Most of the patients presented clinical manifestations of hypercortisolism, (described as Cushing syndrome in **Supplementary Table 1**), frequently accompanied by additional complications (muscle weakness, hypokalemia, hyperglycemia, hypertension, fatigue, and osteoporosis). Other presentations included locoregional MTC effects, most often neck mass or cervical lymphadenopathy.

MTC stage at presentation was reported in 90/99 cases (90.9%). Distant metastases were present in 48/90 patients (53.3%), locally advanced disease in 19/90 patients (21.1%), regional lymph node metastases in 21/90 patients (23.3%), and probable metastatic disease in 2/90 patients (2.2%). Staging was unavailable in 9/99 cases (9.1%). The interval between MTC and ECS diagnosis was documented in 89/99 cases (89.9%): concomitant in 45/89 patients (50.6%), MTC preceded ECS in 43/89 patients (48.3%), median delay 42 months, range 0.5–288 months), and ECS preceded MTC in 1/89 patients (1.1%). Timing was not reported in 10/99 cases (10.1%) (**Table 1**).

**Figure 2:**
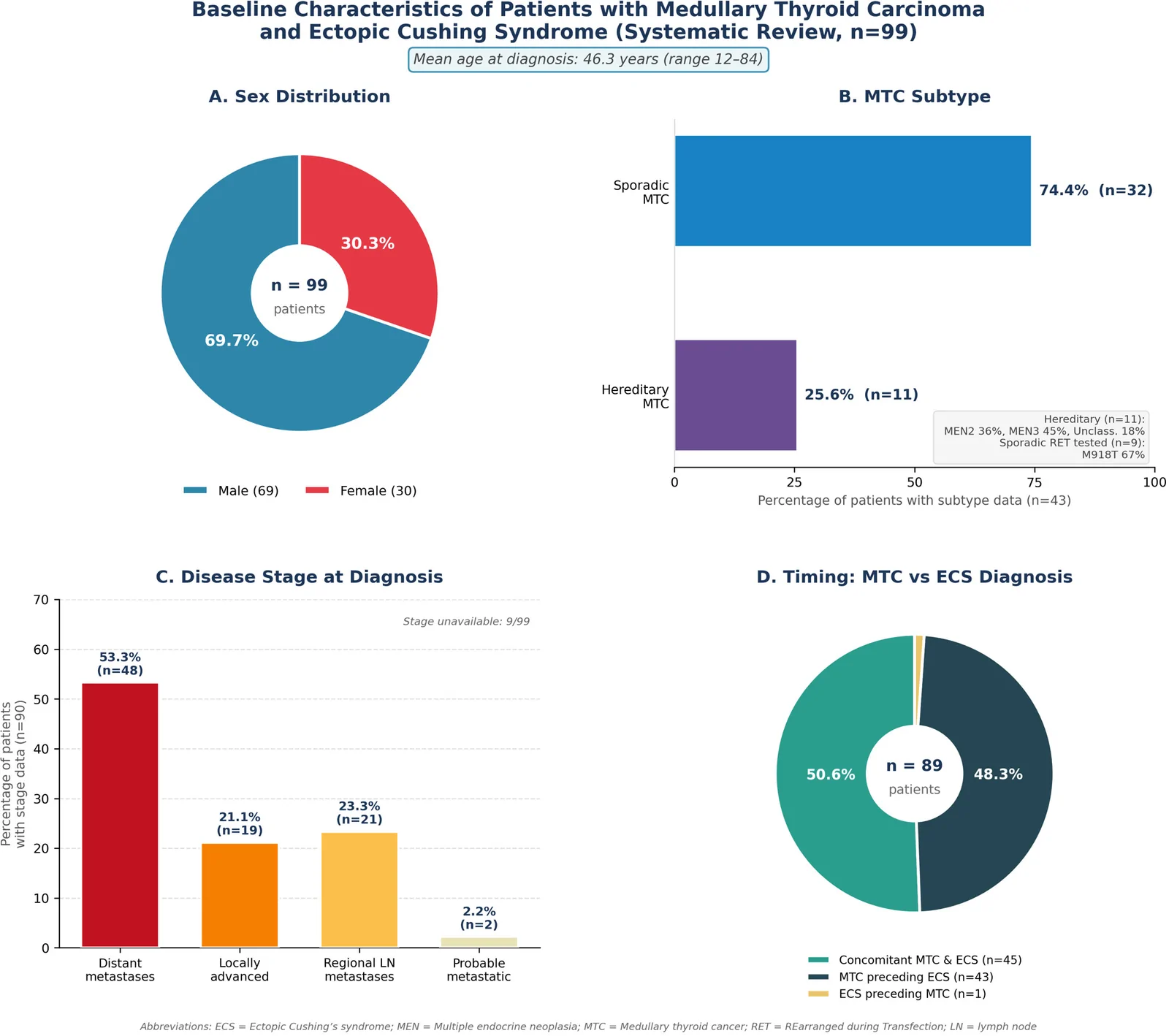
Baseline characteristics of patients with medullary thyroid carcinoma and ectopic Cushing syndrome (systematic review, n=99). **(A)** Sex distribution of the study population. **(B)** Distribution of MTC subtypes among patients with available data. **(C)** Disease stage at diagnosis among patients with available data. **(D)** Temporal relationship between MTC and ECS diagnoses.

**Figure 2** represents a 4-panel graphical summary of **Table 1**.

Any treatment applied for MTC was reported in 77/99 cases (77.8%). Surgery was the initial treatment applied in 62/77 (80.5%), with total thyroidectomy (± regional lymph node dissection) performed in 54/62 (87.1%), and less extensive neck surgery in 8/62 (12.9%). Metastases directed interventions were reported in 10/77 cases (13%). These included metastasectomy in 8/77 cases (10.4%), hepatic chemoembolization in one patient, and radiofrequency ablation in one patient. Adjuvant or palliative therapies included: neck external radiotherapy reported in 14/77 cases (18.2%) and conventional chemotherapy in 12/77 cases (15.6%) (**Table 2**).

**Table 2:** Therapeutic strategies in patients with medullary thyroid carcinoma and ectopic Cushing syndrome (systematic review, n=99).

Therapeutic strategies	No. (%)
MTC-directed treatment	
MTC treatment reported	77/99 (77.8%)
Surgical intervention:	62/77 (80.5%)
TT ± RLND	54/62 (87.1%)
Less extensive neck surgery	8/62 (12.9%)
Metastasis directed interventions:	10/77 (13%)
Metastasectomy	8/77 (10.4%)
Hepatic chemoembolization	1/77 (1.3%)
Hepatic radiofrequency ablation	1/77 (1.3%)
Neck external radiotherapy	14/77 (18.2%)
Conventional chemotherapy	12/77 (15.6%)
Treatment with TKIs	22/77 (28.6%)
Multi-targeted TKIs:	
Vandetanib	15
Cabozantinib	4
Sorafenib	3
Sunitinib	2
Selective RET inhibitors:	
Selpercatinib	5
HS-10365*	1
Management of hypercortisolism	
Hypercortisolism treatment reported	79/99 (77.8%)
Bilateral adrenalectomy	33/79 (41.8%)
Steroidogenesis inhibitors:	
Ketoconazole	20
Metyrapone	16
Mitotane	8
Aminoglutethimide	4
Osilodrostat	1
Etomidate	1
Glucocorticoid receptor blocker, mifepristone	2
Somatostatin analogues^a^	11

MTC, medullary thyroid cancer; RET, REarranged during Transfection proto-oncogene; RLND, regional lymph node dissection (distinction between central and latero-cervical compartment lymphadenectomy in most studies was not available); TT, total thyroidectomy; TKIs, tyrosine kinase inhibitors.

*Protocol HS-10365-101; Jiangsu Haosun Pharmaceutical Group Co., Ltd.). ^a^ Somatostatin analogues were grouped separately because their therapeutic purpose varied across reports (tumor control, cortisol suppression, diarrhea control).

TKIs were administered in 22/77 reported cases (28.6%), with the earliest use in the literature reported in 2013. Multi-targeted tyrosine kinase inhibitors (MKIs) included vandetanib (n=15), cabozantinib (n=4), sorafenib (n=3), and sunitinib (n=2). Selective RET inhibitors were used in 6/77 cases (7.8%), of which selpercatinib (n=5) and the selective RET inhibitor HS-10365 (n=1; protocol HS-10365-101; Jiangsu Haosun Pharmaceutical Group Co., Ltd.) (**Table 2**).

Management of hypercortisolism was reported in 79/99 cases (77.8%) and was heterogeneous, requiring combination therapeutic strategies. In 33/79 cases (41.8%) BLA was eventually required. Interestingly, in 21 out of 33 patients, BLA was used as first-line treatment for CS, while for the remaining 12 patients it was a rescue treatment after failure of medical therapy. In the letter cases, prior medical regiments included sequential or combination treatments with steroidogenesis inhibitors (ketoconazole in 8 cases, aminoglutethimide in 3 cases, metyrapone 2 cases, mitotane in 3 cases), SSA (3 cases) and TKI (one case) **Supplementary Table 2**.

Across the literature cases, medical therapies for hypercortisolism primarily consisted of steroidogenesis inhibitors: ketoconazole (n=20), metyrapone (n=16), mitotane (n=8), aminoglutethimide (n=4), osilodrostat (n=1), and etomidate (n=1). Additionally, the glucocorticoid receptor blocker, mifepristone was used in 2 cases (**Table 2**).

Somatostatin analogues (SSA) were used in 11 cases, with overlapping therapeutic purposes. Across the included cases, SSA were administered as part of tumor directed therapy, as an attempt to suppress hypercortisolism, or adjunctively for symptom control, particularly secretory diarrhea (**Table 2**).

**Figure 3:**
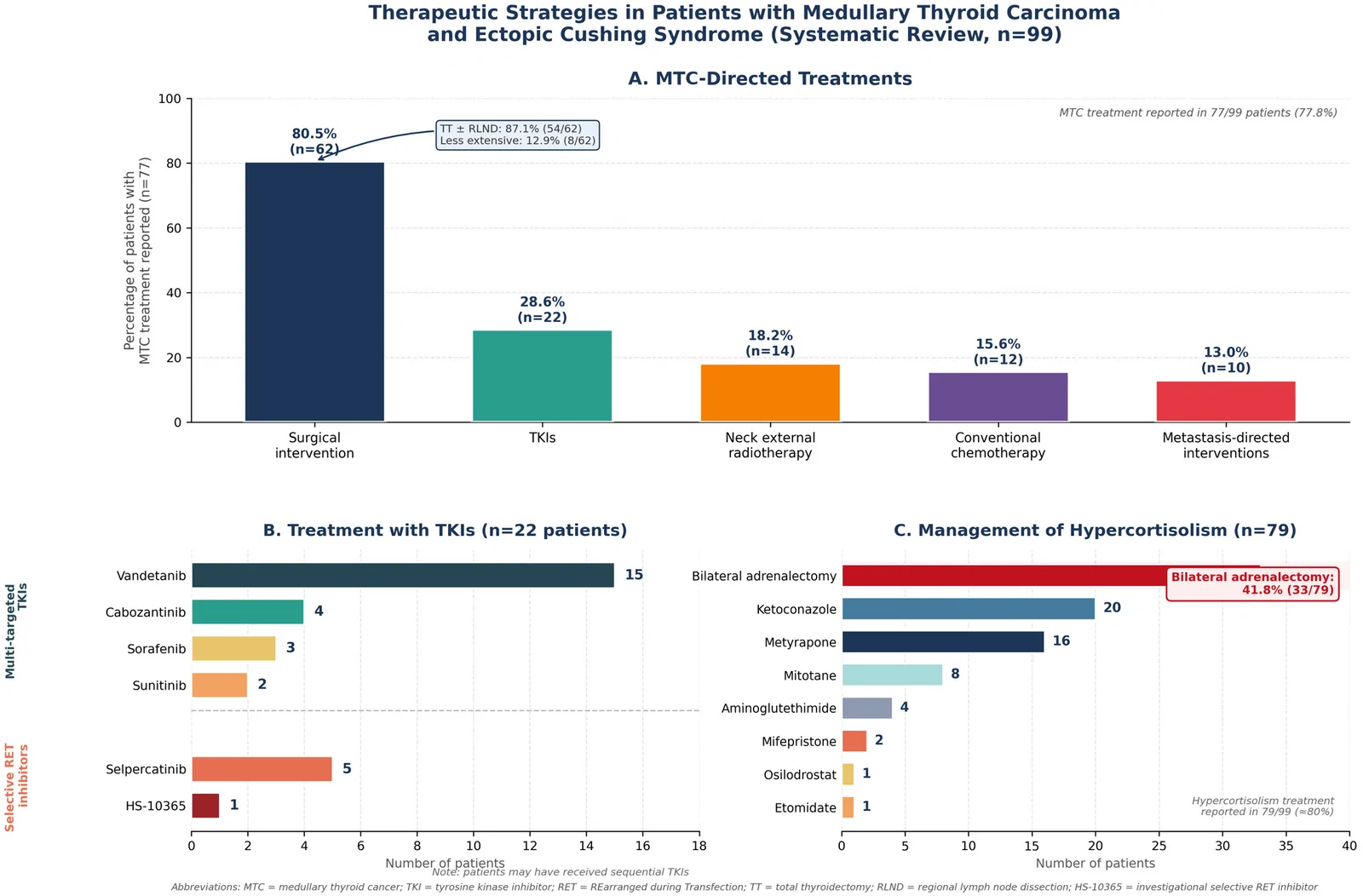
Therapeutic strategies in patients with medullary thyroid carcinoma and ectopic Cushing syndrome (systematic review, n=99). **(A)** MTC-directed treatments among patients with reported treatment data (n=77). **(B)** Distribution of TKI therapies among the patients treated accordingly (n=22). **(C)** Management of hypercortisolism among patients with reported treatment data (n = 79).

**Figure 3** represents a 3-panel graphical summary of **Table 2**.

Follow-up data were reported in 46/99 cases (46.5%), ranging from a few days to 291 months. Causes of death were documented in 39/99 cases (39.4%), predominantly attributable to either complications of uncontrolled ECS (respiratory infections, sepsis, peritonitis secondary to gastrointestinal perforations, and opportunistic infections such as meningitis), or MTC progression **Supplementary Table 2**.

### Additional institutional cases

The four additional cases demonstrated a predominantly middle-aged presentation, with a median age of 53 years (range 20-70), and three of the four patients (75%) were male. Genetic testing identified sporadic disease in two patients, including one harboring a RET M918T mutation, while the remaining cases were classified as probably sporadic based on personal and familial history. Disease burden at presentation was substantial: three patients had metastatic MTC and one with locally advanced disease. Regarding MTC treatment all patients underwent thyroid surgery. After surgery, two patients subsequently received vandetanib, with biochemical improvement of calcitonin and CEA in both cases and morphological outcome of stable disease (Case 1) and partial response (Case 2). In case 3, adjuvant external radiotherapy of the residual mediastinum mass was used without biochemical or morphological improvement (**Table 3**).

**Table 3:** Management of medullary thyroid carcinoma and ectopic Cushing syndrome across additional four institutional cases.

Case	MTC stage at diagnosis	Cortisol burden at presentation	Cortisol-lowering therapy	Bilateral adrenalectomy	Biochemical control of hypercortisolism	Tumor directed treatment	Calcitonin (baseline → post-therapy)	CEA (baseline → post-therapy)	Morphologic tumor response^*^
1	Metastatic	S-cortisol 1600 ACTH 156	NA (recommended but not administered)	Not performed (frailty, progressive deterioration)	Not achieved	Cytoreductive TT + RLND → vandetanib	177,000 → 82,600 (post-op) → 43,900 (12 mo vandetanib)	715 → NA	Stable disease
2	Locally advanced	S-cortisol 877 UFC >1590 ACTH 115	NA	Not indicated	Achieved (cortisol normalized post-TT)	Cytoreducetive TT→ vandetanib	1,815 → 1,290 (post-op) → 37.8 (6 mo vandetanib)	97 → 38 (post-op) → 9.39 (6 mo vandetanib)	Partial response
3	Metastatic	NA (post-adrenalectomy)	NA	Performed prior to presentation	Not achieved (ACTH rose; persistent ectopic drive)	TT + mediastinal mass resection → mediastinum external radiation	>900 → >1,200	NA	Progressive disease
4	Metastatic	S-cortisol 1,374 ACTH 154 no suppression at HDDST	Ketoconazole (up to 800 mg/day; ineffective)	Performed as rescue therapy	Not achieved	Cytoreductive TT	>2,000 → >2,000	NA	Progressive disease

ACTH, adrenocorticotropic hormone; CEA, carcinoembryonic antigen; EBRT, external-beam radiotherapy; HDDST, high dose dexamethasone suppression test; mo, months; NA, not available/not applicable; RLND, regional lymph node; S- cortisol, morning serum cortisol; TT, total thyroidectomy, UFC, urinary free cortisol. ^*^Tumor response was assessed according to RECIST criteria 1.1.

Management of hypercortisolism across the four institutional cases was heterogeneous. BLA was performed in two patients: in case 3 as prior management before presentation, and in case 4 as rescue therapy after failure of ketoconazole. Among the two cases who did not undergo BLA, case 1 did not achieve hypercortisolism control despite thyroid surgery and vandetanib treatment; cortisol-lowering medical therapy was recommended but ultimately not administered. In contrast, case 2 achieved normalization of cortisol following total thyroidectomy, and no further hypercortisolism-directed treatment was required (**Table 3**).

## Discussion

ECS secondary to MTC is an exceptionally rare paraneoplastic manifestation, occurring in approximately 0.7% of MTC cases (7). Through published reports from 1959 to January 2026, our systematic review identified 99 cases; separately, we have considered four institutional cases. Its rarity should also be interpreted in the context of potential under recognition, as routine cortisol assessment is not part of MTC follow-up and early or mild ECS may go undetected.

In the published cases, we observed a male predominance (69.7%) and with a mean age at diagnosis of 46.3 years (range 12–84 years). Although reporting bias cannot be excluded, the consistent male predominance and younger age at presentation across published cases suggest that sex- and age-related factors may influence the development or clinical recognition of ECS due to MTC. This pattern aligns with our institutional series. Evidence also indicates that MTC follows a more aggressive behavior in males, although the underlying mechanism remains unclear (95).

Most patients with available data had sporadic MTC (32/43, 74.4%), mirroring the known epidemiology of the disease (96). This distribution indicates that ECS is not disproportionately linked to hereditary forms but rather arises in the context of advanced disease, which is more typical of sporadic MTC due to delayed diagnosis and limited routine RET mutation screening. Among sporadic tumors, the RET M918T somatic mutation predominated (6/9 cases tested, 66.7%), consistent with its association with aggressive and metastatic disease. A similar pattern was seen in our institutional cases, where both patients tested for germline RET mutations had sporadic disease and one carried an M918T somatic mutation. However, no connection has been established between RET signaling pathways and POMC gene expression or ectopic ACTH production, suggesting that this association reflects tumor burden rather than RET-specific functional effects.

A high proportion of patients presented with metastatic MTC at diagnosis. This finding closely aligns with *Corsello* et al. (83), who reported distant metastases in 51% (44/86) of cases with available staging, compared with 53.3% (48/90) in our cohort. This was also reflected in our institutional series, where three of four patients had distant metastases. Such consistency across studies reinforces that clinically overt ECS is strongly associated with disseminated MTC, occurring predominantly in the context of metastatic rather than localized disease.

The clinical presentation of MTC-associated ECS was dominated by overt hypercortisolism and its complications, consistent with prior reports in which cortisol symptoms overshadowed locoregional thyroid symptoms (83). This creates a diagnostic challenge, as severe ACTH-dependent hypercortisolism should raise suspicion for MTC even in the absence of a palpable thyroid lesion, and early calcitonin, CEA, and cervical ultrasound may facilitate detection. In metastatic MTC treated with TKIs, hypercortisolism comorbidities can further exacerbate drug-related toxicities, including QTc prolongation and arrhythmias (96, 99).

The temporal relationship between MTC and ECS showed that most cases were concomitant (50.6%), while MTC preceded ECS in 48.3% (median delay 42 months). ECS preceded MTC in only one reported case. This pattern likely reflects the need for substantial tumor burden to produce clinically significant ACTH or CRH, combined with diagnostic bias: MTC is often detected early through calcitonin screening or neck mass evaluation, whereas hypercortisolism typically becomes clinically evident only when severe.

Management of MTC-associated ECS requires simultaneous control of both the underlying malignancy and hypercortisolism. Although curative intent thyroid surgery remains the first-line therapy, most patients present with unresectable or rapidly progressive disease, necessitating systemic treatment. Steroidogenesis inhibitors often provided incomplete or transient biochemical control, and approximately 41.8% of patients in our review ultimately required BLA for definitive management. The proportion was slightly lower than the 48% reported by *Corsello* et al. (83). The difference may reflect increasing use of TKIs in contemporary reports.

MKIs such as vandetanib and cabozantinib are established therapies for progressive, advanced MTC that have demonstrated efficacy in controlling both tumor progression and hypercortisolism.

In our review, 16 patients received MKIs (patient no. 59, 62, 67, 70–72, 75, 77, 80, 82, 85, 87, 88, 92, 93, 99 from **Supplementary Table 2**): 12 were treated with vandetanib alone, 3 received both vandetanib and cabozantinib, and 1 received cabozantinib only. Half of these patients (8/16) had evaluable paired radiologic and biochemical outcomes. Among these, 4/8 patients (patient no. 71, 77, 87, 92 from **Supplementary Table 2**) achieved both tumor response and biochemical improvement of hypercortisolism, whereas the remaining 50% (patient no. 59, 62, 67, 72 from **Supplementary Table 2**) demonstrated biochemical improvement despite absent radiologic tumor shrinkage. A similar pattern was observed in our institutional series: of the two patients treated with vandetanib, one achieved both biochemical and structural response, whereas the other had stable disease without cortisol improvement. These findings indicate that endocrine remission may occur independently of tumor response and highlight the need of bilateral adrenalectomy when metastatic MTC remains biochemically active despite adequate oncologic therapy.

However, their clinical utility is limited by significant toxicity, which may impair quality of life (97). More recently, selective RET inhibitors have transformed the therapeutic landscape for RET-mutant MTC. Selpercatinib, approved by FDA in 2020, offers superior efficacy and a more favorable safety profile compared to MKIs (98).

In the published literature, we identified only five cases of MTC-associated ECS treated with selpercatinib (12–14, 80, 84). Four cases were previously treated with MKIs: three progressed on vandetanib (12, 13, 80), and one was switched from vandetanib to selpercatinib after identification of a RET mutation (14).

Clinical improvement was rapid and important. *Sitbon* et al. (84) reported resolutions of chronic diarrhea within days, accompanied by improvement in hypercortisolism-related manifestations (muscle strength, skin changes, and fatigue). *Ragnarsson et al* (12). described complete normalization of all cushingoid features by six months. All five cases demonstrated biochemical improvement of hypercortisolism, with two achieving complete biochemical remission (80, 84); notably, urinary free cortisol normalized within two months in one patient despite subtherapeutic dosing (84). Calcitonin and CEA decrease in all five cases, with the earliest reduction documented in one week (14). Biochemical improvement mirrored the radiologic response, with all patients showing lesion shrinkage without new sites of disease, including early reduction by one month and durable responses extending up to 30 months (14, 84). Collectively, these cases highlight selpercatinib as a highly effective option for managing both hypercortisolism and tumor burden in MTC-associated ECS.

In the five reported cases of ECS resolution with selpercatinib, adverse effects were mild, limited primarily to grade 1 headache. Adrenal insufficiency (AI) was documented in four cases despite no prior adrenalectomy (12, 13, 80, 84). Given selpercatinib’s high selectivity for RET with negligible VEGF inhibition, direct adrenal toxicity is unlikely. Instead, the observed AI is best explained by secondary adrenal insufficiency due to hypothalamic–pituitary–adrenal axis suppression from longstanding hypercortisolism. In this context, the development of secondary AI represents a biochemical marker of successful ECS remission rather than an adverse effect attributable to selpercatinib. Routine adrenal function monitoring and glucocorticoid replacement are therefore recommended during the early stages of treatment.

Identification of somatic RET mutations is essential for precision management of MTC complicated by ECS. All five cases treated with selpercatinib harbored the RET M918T somatic mutation (12–14, 80, 84). This observation supports current recommendations for universal germline RET testing and somatic testing when targeted therapy is being considered (96, 99, 100).

This systematic review provides one of the most comprehensive and up-to-date synthesis of ECS secondary to MTC. Nonetheless, important limitations must be acknowledged. First, the evidence base consists almost entirely of case reports and small series, which inherently limits generalizability and introduces substantial publication and selection bias. Second, diagnostic and therapeutic approaches have evolved over the past decades, complicating comparisons across time periods. Third, documentation of ECS diagnostic criteria and severity was highly inconsistent. Many reports lacked complete biochemical evaluation or detailed imaging, making it difficult to uniformly assess disease burden or compare severity across cases. Fourth, treatment reporting was heterogeneous restricting conclusions regarding therapeutic efficacy. Finally, comparisons between our institutional case series and published cases should be interpreted cautiously, as differences in reporting detail, diagnostic workup, and treatment availability inherently limit direct comparability.

## Conclusion

MTC-associated ECS remains an exceptionally rare and highly morbid complication. Through our systematic search, we identified 99 published cases meeting predefined eligibility criteria. Most patients presented with metastatic disease, and prognosis was frequently driven by severe hypercortisolism complications or progressive malignancy. Although rapid control of hypercortisolism is essential, conventional steroidogenesis inhibitors often provide incomplete or transient benefit, leading many patients to require bilateral adrenalectomy for definitive management.

Emerging evidence from recent case reports indicates that selective RET inhibitors, particularly selpercatinib, may offer meaningful dual benefit by achieving both biochemical remission of ectopic hypercortisolism and sustained tumor control in RET-mutated MTC. However, the mechanisms underlying these responses and the broader therapeutic applicability of these agents remain incompletely understood. Prospective multicenter registries and dedicated clinical studies are needed to define optimal treatment sequencing, long-term efficacy, and safety of precision RET-targeted therapies in this rare but clinically severe presentation of MTC.

Looking into the data related with the four additional cases taken from our center, we can conclude that these cases do correlate with those included in the systematic review.

## Data Availability

The datasets presented in this study can be found in online repository: https://doi.org/10.5281/zenodo.22100434.
