# Supplementary material for: Management of ectopic Cushing syndrome in medullary thyroid carcinoma: from case reports to systematic literature review
Source: Front Endocrinol (Lausanne). 2026 Sep 9;17:1933394. doi: 10.3389/fendo.2026.1933394 (PMC13597265; doi:10.3389/fendo.2026.1933394)
Supplement: Supplementary file 1 [file Table1.docx]

**Supplementary Table S1**. Clinical Characteristics and RET Genetic findings in Patients with Medullary Thyroid Carcinoma and Ectopic Cushing Syndrome

| **Patients** | **Age at presentation**  **(years)** | **Sex**  **(M/F)** | **MTC subtype/ RET mutation** | | **Presentation** | **MTC stage at diagnosis** | | **Reference** |
| --- | --- | --- | --- | --- | --- | --- | --- | --- |
| 1. | 19 | F | NA/NA | Back pain | | | Metastatic | Dyson et al. 1959 |
| 2. | NA | M | NA/NA | Confusion | | | Metastatic | Goldberg and McNeil 1967 |
| 3. | 46 | F | Probably hereditary/NA | Weakness | | | Metastatic | Williams et al. 1968 |
| 4. | 49 | F | NA/NA | Diarrhea | | | NA | Williams et al. 1968 |
| 5. | 33 | M | NA/NA | Abdominal pain, diarrhea | | | Locally advanced | Donahower et al. 1968 |
| 6. | 27 | F | NA/NA | Neck mass | | | Locally advanced | Donahower et al. 1968 |
| 7. | 39 | M | NA/NA | CS | | | Metastatic | Szijj et al. 1969 |
| 8. | 53 | F | NA/NA | Neck mass | | | Locally advanced | Melvin et al. 1970 |
| 9. | 61 | F | NA/NA | Neck mass | | | NA | Croughs et al. 1972 |
| 10. | 57 | M | NA/NA | NA | | | NA | Ratcliffe et al. 1972 |
| 11. | 12 | M | Probably sporadic/NA | Neck mass | | | Metastatic | Bordi et al. 1972,  Bussolati et al. 1973 |
| 12. | 54 | M | NA/NA | Diarrhea | | | Locally advanced | Hajjar et al. 1975 |
| 13. | 45 | F | NA/NA | CS | | | Metastatic | Birkenhager et al 1976 |
| 14. | 54 | M | Probably sporadic/NA | NA | | | NA | Kameya et al. 1977 |
| 15. | 48 | F | Probably sporadic/NA | NA | | | NA | Kameya et al. 1977 |
| 16. | 40 | M | Probably sporadic/NA | Neck mass, diarrhea | | | Probably metastatic¹ | Keusch et al. 1977 |
| 17. | 54 | M | NA/NA | Neck mass | | | Metastatic | Takai et al. 1977  Kakudo et al. 1982 |
| 18. | 38 | M | Probably sporadic/NA | CS, diarrhea | | | Metastatic | Muller et al. 1977 |
| 19. | 64 | F | NA/NA | Osteoporosis | | | Metastatic | Rosenberg et al. 1978 |
| 20. | 24 | M | NA/NA | Neck mass | | | Locally advanced | McElduff et al. 1979 |
| 21. | 34 | F | NA/NA | Osteoporosis | | | Locally advanced | Jolivet et al. 1980 |
| 22. | 48 | M | NA/NA | Neck mass | | | N+ | Chin et al. 1981 |
| 23. | 33 | F | NA/NA | NA | | | NA | Charpin et al. 1982 |
| 24. | 57 | M | NA/NA | NA | | | Metastatic | Davies et al. 1982 |
| 25. | 54 | M | NA/NA | Muscle weakness | | | Metastatic | Shimatsu et al. 1983 |
| 26. | 64 | F | NA/NA | Muscle weakness | | | N+ | Belsky et al. 1985 |
| 27. | 59 | F | NA/NA | Dysphagia, diarrhea | | | N+ | Lind et al. 1985 |
| 28. | 62 | M | NA/NA | Neck mass, diarrhea | | | Locally advanced | Ahlman and Tisell 1987 |
| 29. | 58 | M | NA/NA | NA | | | NA | Tourniaire et al. 1988 |
| 30. | 51 | F | NA/NA | Neck mass | | | N+ | Blunt et al. 1989 |
| 31. | 45 | M | NA/NA | Polyuria/polydipsia | | | Metastatic | Popovic et al. 1991 |
| 32. | 32 | M | NA/NA | CS | | | Metastatic | Hijazi et al. 1992 |
| 33. | 45 | M | Hereditary/NA | NA | | | Locally advanced | Mure et al. 1995 |
| 34. | 34 | F | Sporadic/NA | NA | | | Locally advanced | Mure et al. 1995 |
| 35. | 39 | F | Sporadic/NA | NA | | | Locally advanced | Mure et al. 1995 |
| 36. | 36 | F | Sporadic/NA | NA | | | Locally advanced | Mure et al. 1995 |
| 37. | 84 | M | NA/NA | NA | | | Metastatic | Winquist et al. 1995 |
| 38. | 20 | F | MEN3/NA | Tongue and eyelid mucosal  neuromas, thyroid  enlargement, CS | | | N+ | Tagliabue et al. 1996 |
| 39. | 62 | F | NA/NA | Diarrhea, fatigue | | | Metastatic | Oates et al. 2000 |
| 40. | 47 | M | MEN2/NA | CS | | | Locally advanced | von Mach et al. 2002 |
| 41. | 55 | M | Sporadic/NA | CS, hypertension | | | Metastatic | Kristiansen et al 2002 |
| 42. | 38 | M | MEN2/618 | CS | | | Locally advanced | Smallridge et al. 2003 |
| 43. | 40 | M | Sporadic/NA | NA | | | Metastatic | Barbosa et al. 2005 |
| 44. | 52 | M | Sporadic/NA | NA | | | Metastatic | Barbosa et al. 2005 |
| 45. | 41 | M | Sporadic/NA | CS | | | Metastatic | Barbosa et al. 2005 |
| 46. | 68 | M | Sporadic/NA | NA | | | Metastatic | Barbosa et al. 2005 |
| 47. | 35 | F | Sporadic/NA | NA | | | Metastatic | Barbosa et al. 2005 |
| 48. | 45 | M | Hereditary/NA | NA | | | Metastatic | Barbosa et al. 2005 |
| 49. | 41 | M | Sporadic/NA | NA | | | Metastatic | Barbosa et al. 2005 |
| 50. | 48 | M | Sporadic/NA | CS | | | Locally advanced | Barbosa et al. 2005 |
| 51. | 30 | M | Sporadic/NA | CS | | | Metastatic | Barbosa et al. 2005 |
| 52. | 75 | M | Sporadic/NA | NA | | | N+ | Barbosa et al. 2005 |
| 53. | 51 | M | Probably sporadic/NA | Neck mass | | | Metastatic | Bourlet et al. 2007 |
| 54. | 42 | M | Sporadic/NA | CS | | | NA | Sand et al. 2007 |
| 55. | 45 | M | Sporadic/NA | CS | | | N+ | Chrisoulidou et al. 2008 |
| 56. | 51 | F | MEN2/609 | CS | | | Metastatic | Zaydfudim et al. 2008 |
| 57. | 13 | M | MEN3/NA | Growth retardation, CS, proximal myopathy | | | N+ | Dhanwal et al. 2010 |
| 58. | 49 | M | Probably sporadic/NA | CS | | | Locally advanced | Wang et al. 2011 |
| 59. | 58 | M | NA/NA | Depression, muscle weakness, diarrhea | | | N+ | Baudry et al. 2013 |
| 60. | 31 | F | MEN3/NA | Hyperpigmentation | | | Locally advanced | Kurozumi et al. 2013 |
| 61. | 45 | M | Sporadic/ M918T | CS | | | Metastatic | Barroso-Sousa et al. 2014 |
| 62. | 17 | M | MEN3/ M918T | Neck mass, diarrhea | | | Metastatic | Fox et al. 2013; Nella et al. 2014 |
| 63. | 48 | M | Sporadic/NA | MTC | | | N+ | Choi et al. 2014 |
| 64. | 13 | M | MEN3/ M918T | Growth deceleration, weight gain | | | Metastatic | Singer et al. 2014 |
| 65. | 30 | M | Sporadic/NA | Diabetes insipidus, hypokalemia, hyperglycemia | | | N+ | Hammami et al. 2015 |
| 66. | 64 | M | Sporadic/NA | CS | | | N+ | Marques et al. 2015 |
| 67. | 37 | F | Sporadic/NA | Fatigue, dyspnea | | | N+ | Pitoia et al. 2015 |
| 68. | 51 | M | NA/NA | Hyperglycemia, hypokalemia | | | Metastatic | Verburg et al. 2015 |
| 69. | 72 | M | MEN2/ C618Y/ R844Q | Hypertensive crisis, muscle weakness, hyperpigmented, weight and body hair loss | | | Metastatic | Ares et al. 2016 |
| 70. | 67 | M | NA/NA | Diarrhea, muscle weakness | | | Metastatic | Matheny et al. 2016 |
| 71. | 55 | M | Sporadic/NA | MTC | | | N+ | Bseiso et al. 2016 |
| 72. | 58 | M | Sporadic/ M918T | Diarrhea, muscle weakness, depression | | | Metastatic | Paepegaey et al. 2017 |
| 73. | 42 | F | NA/NA | NA | | | Metastatic | Sathyakumar et al. 2017 |
| 74. | 18 | M | NA/NA | NA | | | Metastatic | Sathyakumar et al. 2017 |
| 75. | 35 | M | Sporadic/NA | Supraclavicular LN | | | N+ | Wijewardene et al. 2017 |
| 76. | 53 | M | NA/NA | CS | | | Metastatic | Wijewardene et al. 2017 |
| 77. | 38 | M | Sporadic/NA | Neck mass | | | Metastatic | Wijewardene et al. 2017 |
| 78. | 62 | M | NA/NA | CS, thyroid mass | | | Metastatic | Parihar et al. 2018 |
| 79. | 54 | M | NA/NA | Facial plethora, neck mass | | | N+ | Ferreira et al. 2019 |
| 80. | 74 | F | Sporadic/„missense mutation” | Diarrhea, weight loss | | | Metastatic | Pivovarova et al. 2019 |
| 81. | 61 | F | NA/NA | MTC, hypokalemia, dyspnea, and acute abdominal pain from diverticular rupture | | | Metastatic | Shahidi et al. 2019 |
| 82. | 41 | M | Sporadic/ M918T | Back pain | | | N+ | Forde et al. 2021 |
| 83. | 28 | F | NA/NA | NA | | | Probably metastatic² | Lopez-Montoya et al. 2021 |
| 84. | 22 | F | Probably sporadic/NA | Autopsy | | | Locally advanced | Cheong et al.2021 |
| 85. | 32 | M | Sporadic/Neg | Fatigue, muscle weakness, weight loss | | | Metastatic | Corsello et al. 2022 |
| 86. | 49 | F | Sporadic/ M918T | MTC | | | Metastatic | Sitbon et al. 2022 |
| 87. | 65 | M | Probably sporadic/NA | CS | | | Metastatic | Mateen et al. 2022 |
| 88. | 62 | M | Sporadic/Exon 16 | CS | | | Metastatic | Ragnarsson et al. 2022 |
| 89. | 69 | M | Sporadic/NA | CS | | | N+ | Mäkinen et al. 2023 |
| 90. | 71 | M | NA/NA | NA | | | Metastatic | Dormoy et al. 2023 |
| 91. | 67 | F | Probably sporadic/NA | Severe hypokalemia | | | Locally advanced | Irimie et al. 2023 |
| 92. | 37 | M | Sporadic/ M918T | CS | | | Metastatic | Jazdarehee A et al. 2024 |
| 93. | 45 | M | NA/NA | CS | | | Locally advanced | Komisarz-Calik et al. 2024 |
| 94. | 23 | M | NA/NA | CS | | | N+ | Alperen Onur et al.2024 |
| 95. | 30 | M | NA/NA† | CS and neck mass | | | NA† | Jha et al. 2024 |
| 96. | 52 | M | Probably sporadic/NA | CS | | | N+ | Oweidat et al. 2025 |
| 97. | 39 | M | NA/NA | CS and neck mass | | | Metastatic | Timofeeva et al 2025 |
| 98. | 55 | M | Probably MEN2/NA | Neck mass | | | N+ | Wang et al. 2025 |
| 99. | 65 | M | Sporadic/M918T | Neck mass | | | Metastatic | Seguchi et al. 2025 |

Abbreviations: CS, Cushing’s syndrome; F, female; M, male; MEN, multiple endocrine neoplasia; MTC, medullary thyroid cancer; N+, lymph node metastases; NA, not available.

Probably sporadic = no data regarding RET mutations testing but personal/familial history suggestive for sporadic disease. Probably hereditary = no data regarding RET mutations testing but personal/familial history suggestive for hereditary disease. Probably metastatic - insufficient primary source detail confirming distant metastases at diagnosis; ¹, Keusch et al. (1977) document lung and liver metastases only at autopsy years after initial MTC diagnosis (1968), precluding determination of stage at presentation; ², Lopez-Montoya et. al. (2021) report “advanced medullary thyroid carcinoma” in text and “metastatic” in table without imaging or histopathologic corroboration of M1 disease.

˝-˝ Negative; † Case included based on published abstract data meeting inclusion criteria (confirmed medullary thyroid carcinoma and ectopic Cushing syndrome), detailed information limited to abstract, so RET mutation status and MTC stage at diagnosis were not available.
