# Supplementary material for: Management of ectopic Cushing syndrome in medullary thyroid carcinoma: from case reports to systematic literature review
Source: Front Endocrinol (Lausanne). 2026 Sep 9;17:1933394. doi: 10.3389/fendo.2026.1933394 (PMC13597265; doi:10.3389/fendo.2026.1933394)
Supplement: Supplementary file 2 [file Table2.docx]

**Supplementary Table S2** Treatment Management, Time Between Diagnosis, and Follow-up of Patients with Medullary Thyroid Carcinoma and Ectopic Cushing Syndrome.

| **Patients** | **Delay MTC–ECS (months)** | **Treatment of MTC** | **Treatment of CS** | **Follow-up**  **(Months)** | **Cause of death** | **Reference** |
| --- | --- | --- | --- | --- | --- | --- |
| 1. | Concomitant | None | BLA | 1 | Cryptococcal meningitis | Dyson et al. 1959 (16) |
| 2. | Concomitant | Hemithyroidectomy | BLA | 24 | MTC | Goldberg and McNeil 1967 (17) |
| 3. | Concomitant | None | None | NA | During surgery | Williams et al. 1968 (18) |
| 4. | 12 (MTC before CS) | Hemithyroidectomy | None | 18 | NA | Williams et al. 1968 (18) |
| 5. | ~48 (MTC before CS) | TT + RLND | BLA | 10 | Diverticulitis perforation | Donahower et al. 1968 (19) |
| 6. | Concomitant | TT + RLND | BLA | NA | NA | Donahower et al. 1968 (19) |
| 7. | Concomitant | None | None | 0.6 | Pulmonary edema | Szijj et al. 1969 (20) |
| 8. | Concomitant | TT + RT | TT | NA | NA | Melvin et al. 1970 (21) |
| 9. | 60 (MTC before CS) | RT | BLA | 72 | Bronchopneumonia | Croughs et al. 1972 (22) |
| 10. | 8 (MTC before CS) | NA | NA | 3 | NA | Ratcliffe et al. 1972 (23) |
| 11. | 18 (MTC before CS) | TT + RLND | NA | NA | NA | Bordi et al. 1972 (24)  Bussolati et al. 1973 (25) |
| 12. | 18 (MTC before CS) | TT + RT | BLA | NA | NA | Hajjar et al. 1975 (26) |
| 13. | Concomitant | TT + metastasectomy | TT + metastasectomy | NA | NA | Birkenhager et al 1976 (27) |
| 14. | NA | NA | NA | NA | NA | Kameya et al. 1977 (28) |
| 15. | NA | NA | NA | NA | NA | Kameya et al. 1977 (28) |
| 16. | 36 (MTC before CS) | TT | None | 72 | NA | Keusch et al. 1977 (29) |
| 17. | Concomitant | TT+ RLND | BLA | 31 | NA | Takai et al. 1977 (30)  Kakudo et al. 1982(31) |
| 18. | Concomitant | TT + CHT | None | 2 | MTC | Muller et al. 1977 (32) |
| 19. | Concomitant | Subtotal thyroidectomy | Metyrapone | 2 | Acute intestinal obstruction | Rosenberg et al. 1978 (33) |
| 20. | 108 (MTC before CS) | Hemithyroidectomy + RLND | Metyrapone + aminoglutethimide → adriamycin | NA | NA | McElduff et al. 1979 (34) |
| 21. | Concomitant | TT | TT | NA | NA | Jolivet et al. 1980 (35) |
| 22. | Concomitant | NA | NA | NA | Duodenal ulcer perforation | Chin et al. 1981 (36) |
| 23. | NA | TT | BLA | 36 | NA | Charpin et al. 1982 (37) |
| 24. | Concomitant | Subtotal thyroidectomy | BLA | NA | NA | Davies et al. 1982 (38) |
| 25. | Concomitant | „Neck surgery” + CHT | BLA | NA | NA | Shimatsu et al. 1983 (39) |
| 26. | Concomitant | TT | BLA | 3 | Diverticulitis perforation | Belsky et al. 1985 (40) |
| 27. | 37 (MTC before CS) | „Neck surgery” + CHT | Metyrapone | 42 | Progressive metastatic disease | Lind et al. 1985 (41) |
| 28. | 180 (MTC before CS) | TT + metastasectomy + SSA | BLA | NA | NA | Ahlman and Tisell 1987 (42) |
| 29. | 24 (CS before MTC) | TT | TT | NA | NA | Tourniaire et al. 1988 (43) |
| 30. | 108 (MTC before CS) | TT + RT + CHT | Metyrapone + aminoglutethimide → bilateral adrenal venous ablation → bilateral arterial embolization → BLA | NA | NA | Blunt et al. 1989 (44) |
| 31. | 8 (MTC before CS) | RT of neck → craniotomy for pituitary mass reduction → RT of pituitary + CHT | NA | 13 | NA | Popovic et al. 1991 (45) |
| 32. | Concomitant | TT + RLND + metastasectomy | Ketoconazole + aminoglutethimide + metyrapone → BLA | NA | NA | Hijazi et al. 1992 (46) |
| 33. | 29 (MTC before CS) | TT + RLND + RT + CHT | SSA → ketoconazole + mitotane → BLA | 39 | NA | Mure et al. 1995 (47) |
| 34. | 95 (MTC before CS) | TT + RLND + RT | NA | 95 | Pulmonary disease | Mure et al. 1995 (47) |
| 35. | 20 (MTC before CS) | TT +RLND + CHT | Ketoconazole-> mitotane-> BLA | 42 | Disease progression | Mure et al. 1995 (47) |
| 36. | 2 (MTC before CS) | TT + RLND + CHT | Ketoconazole → SSA → mitotane + aminoglutethimide → BLA | 30 | Brain metastases | Mure et al. 1995 (47) |
| 37. | NA | NA | Ketoconazole | NA | NA | Winquist et al. 1995 (48) |
| 38. | Concomitant | TT + RLND | TT + RLND | 12 | NA | Tagliabue et al. 1996 (49) |
| 39. | Concomitant | SSA → chemoembolization of liver metastases | No treatment | 6 | NA | Oates et al. 2000 (50) |
| 40. | 132 (MTC before CS) | TT | BLA | NA | NA | von Mach et al. 2002 (51) |
| 41. | Concomitant | TT + RLND → metastasectomy | TT + RLND → metastasectomy | NA | NA | Kristiansen et al 2002 (52) |
| 42. | 48 (MTC before CS) | TT + RLND | BLA | NA | NA | Smallridge et al. 2003 (53) |
| 43. | 50 (MTC before CS) | NA | NA | 65 | Peritonitis | Barbosa et al. 2005 (7) |
| 44. | 36 (MTC before CS) | NA | NA | 49 | Peritonitis | Barbosa et al. 2005 (7) |
| 45. | Concomitant | NA | NA | 20 | MTC | Barbosa et al. 2005 (7) |
| 46. | 36 (MTC before CS) | NA | NA | 38 | MTC | Barbosa et al. 2005 (7) |
| 47. | 3 (MTC before CS) | NA | NA | 33 | MTC | Barbosa et al. 2005 (7) |
| 48. | 17 (MTC before CS) | NA | NA | 29 | MTC | Barbosa et al. 2005 (7) |
| 49. | 91 (MTC before CS) | NA | NA | 95 | Peritonitis | Barbosa et al. 2005 (7) |
| 50. | Concomitant | „Neck surgery” | „Neck surgery” | 108 | NA | Barbosa et al. 2005 (7) |
| 51. | Concomitant | NA | „Anticortisolic drugs” | 36 | NA | Barbosa et al. 2005 (7) |
| 52. | 36 (MTC before CS) | NA | NA | 43 | MTC | Barbosa et al. 2005 (7) |
| 53. | 45 (MTC before CS) | TT + RLND + CHT | CHT + mitotane + embolization of hepatic and adrenal metastases → mitotane | NA | NA | Bourlet et al. 2007 (54) |
| 54. | 120 (MTC before CS) | TT + RLND | Ketoconazole → BLA | NA | Respiratory infection and failure | Sand et al. 2007 (55) |
| 55. | 288 (MTC before CS) | NA | Metyrapone | 291 | NA | Chrisoulidou et al. 2008 (56) |
| 56. | Concomitant | CHT | Ketoconazole → BLA | NA | NA | Zaydfudim et al. 2008 (57) |
| 57. | Concomitant | TT + RLND | TT + RLND | NA | NA | Dhanwal et al. 2010 (58) |
| 58. | Concomitant | TT + RLND | NA | NA | NA | Wang et al. 2011 (59) |
| 59. | Concomitant | Vandetanib | Metyrapone + mitotane + SSA + ketoconazole → vandetanib | NA | NA | Baudry et al. 2013 (60) |
| 60. | 240 (MTC before CS) | TT | BLA | NA | NA | Kurozumi et al. 2013 (61) |
| 61. | Concomitant | Sorafenib | Sorafenib | NA | NA | Barroso-Sousa et al. 2014 (62) |
| 62. | Concomitant | TT + RLND + metastasectomy + RT → vandetanib → sunitinib → sorafenib → cabozantinib | Vandetanib → ketoconazole → BLA | 72 | MTC | Fox et al. 2013 (63)  Nella et al. 2014 (64) |
| 63. | 168 (MTC before CS) | TT + RLND + MIBG | Ketoconazole → BLA | NA | NA | Choi et al. 2014 (65) |
| 64. | 33 (MTC before CS) | TT + RLND | BLA | NA | NA | Singer et al. 2014 (66) |
| 65. | 42 (MTC before CS) | TT + RLND + RT → sorafenib | Sorafenib + mifepristone | 43 | Liver failure and sepsis | Hammami et al. 2015 (67) |
| 66. | 96 (MTC before CS) | TT + RLND + RT → sunitinib | Metyrapone → sunitinib | NA | NA | Marques et al. 2015 (68) |
| 67. | 192 (MTC before CS) | TT + RLND → vandetanib | Vandetanib | NA | NA | Pitoia et al. 2015 (69) |
| 68. | 48 (MTC before CS) | NA | SSA → BLA | 49 | Sepsis,bone marrow  infiltration, respiratory  failure | Verburg et al. 2015 (70) |
| 69. | Concomitant | TT + RLND | Ketoconazole + TT + RLND | NA | NA | Ares et al. 2016 (71) |
| 70. | Concomitant | Vandetanib | Ketoconazole → mifepristone → etomidate + vandetanib → BLA | 1 | Pneumonia | Matheny et al. 2016 (6) |
| 71. | 240 (MTC before CS) | TT + RLND → SSA → vandetanib | Metyrapone → vandetanib | NA | NA | Bseiso et al. 2016 (72) |
| 72. | Concomitant | Vandetanib → metastasectomy + RLND | Metyrapone → mitotane → SSA → ketoconazole -→ vandetanib | NA | NA | Paepegaey et al. 2017 (73) |
| 73. | NA | NA | NA | NA | Sepsis | Sathyakumar et al. 2017 (74) |
| 74. | NA | NA | NA | NA | Sepsis | Sathyakumar et al. 2017 (74) |
| 75. | 6 (MTC before CS) | TT + RLND → vandetanib | Ketoconazone →  ketoconazole + vandetanib → vandetanib + mitotane | 18 | Pneumonia | Wijewardene et al. 2017 (75) |
| 76. | Concomitant | TT + RLND + RT | RT + ketoconazole | NA | CS | Wijewardene et al. 2017 (75) |
| 77. | 24 (MTC before CS) | TT + RLND → vandetanib | Ketoconazole → ketoconazole + vandetanib  → ketoconazole + metyrapone → metyrapone + mitotane | 48 | CS | Wijewardene et al. 2017 (75) |
| 78. | Concomitant | TT + RLND | TT + RLND | NA | NA | Parihar et al. 2018 (76) |
| 79. | Concomitant | RT + CHT | Ketoconazole | 6 | NA | Ferreira et al. 2019 (77) |
| 80. | 30 (MTC before CS) | TT → vandetanib | Ketoconazole | 36 | NA | Pivovarova et al. 2019 (78) |
| 81. | NA | NA | NA | NA | NA | Shahidi et al. 2019 (79) |
| 82. | Concomitant | TT + RLND → vandetanib → cabozantinib → selpercatinib | Metyrapone + SSA → vandetanib → cabozantinib → selpercatinib | NA | NA | Forde et al. 2021 (80) |
| 83. | NA | TT + SSA | BLA | 3 | MTC | Lopez-Montoya et al. 2021 (81) |
| 84. | NA | NA | NA | NA | Diabetic ketoacidosis | Cheong et al.2021 (82) |
| 85. | Concomitant | TT + RLND → SSA + vandetanib | BLA | NA | NA | Corsello et al. 2022 (83) |
| 86. | 168 (MTC before CS) | TT + RLND → RFA (liver metastases) + RT and denosumab (bone metastases) → selpercatinib | Right adrenal gland embolization  → ketoconazole → selpercatinib | 204 | NA | Sitbon et al. 2022 (84) |
| 87. | Concomitant | Cabozantinib | Metyrapone | NA | NA | Mateen et al. 2022 (85) |
| 88. | 84 (MTC before CS) | TT → metastasectomy → vandetanib → cabozantinib → selpercatinib → temozolomid → capecitabin | Metyrapone → selpercatinib | NA | NA | Ragnarsson et al. 2022 (12) |
| 89. | Concomitant | TT + RLND | Ketoconazole → BLA | NA | NA | Mäkinen et al. 2023 (86) |
| 90. | NA | NA | Osilodrostat | NA | NA | Dormoy et al. 2023 (87) |
| 91. | Concomitant | NA | BLA | 0.7 | Adrenal crisis | Irimie et al. 2023 (88) |
| 92. | Concomitant | Vandetanib → selpercatinib | Vandetanib → selpercatinib | 23 | NA | Jazdarehee A et al. 2024 (13) |
| 93. | Concomitant | TT → vandetanib | TT → vandetanib | NA | NA | Komisarz-Calik et al. 2024 (89) |
| 94. | Concomitant | TT + RLND | Metyrapone | NA | NA | Alperen Onur et al.2024 (90) |
| 95. | Concomitant | TT → RT | BLA | NA† | NA† | Jha et al. 2024 (91) |
| 96. | Concomitant | TT + RLND | TT + RLND | 0 | Postoperative hemorrhage | Oweidat et al. 2025 (92) |
| 97. | Concomitant | TT + RLND | TT + RLND | NA | NA | Timofeeva et al 2025 (93) |
| 98. | 11 (MTC before CS) | TT + RLND → a Phase I/II, selective RET kinase inhibitor (HS-10365) trial | BLA | NA | NA | Wang et al. 2025 (94) |
| 99. | Concomitant | Vandetanib → selpercatinib | Metyrapone → vandetanib → selpercatinib | NA | NA | Seguchi et al. 2025 (14) |

**Abbreviations:** BLA, Bilateral adrenalectomy CHT, chemotherapy; CS, Cushing’s syndrome; RLND, regional lymph node dissection (distinction between central and latero-cervical compartment lymphadenectomy in most studies was not available); MTC, medullary thyroid cancer; NA, not applicable/not reported in the original publication; RT, radiotherapy; SSA, somatostatin analogues; TT, total thyroidectomy.

**Therapeutic regiments:** ˝+˝ indicates combination therapy; ˝→˝ indicates sequential therapy. † Case included based on published abstract data meeting inclusion criteria (confirmed medullary thyroid carcinoma and ectopic Cushing syndrome); detailed information limited to abstract, so follow-up duration and cause of death not reported.
